# Methods, caveats and the future of large-scale microelectrode recordings in the non-human primate

**DOI:** 10.3389/fnsys.2015.00149

**Published:** 2015-11-03

**Authors:** Nicholas M. Dotson, Baldwin Goodell, Rodrigo F. Salazar, Steven J. Hoffman, Charles M. Gray

**Affiliations:** ^1^Department of Cell Biology and Neuroscience, Montana State UniversityBozeman, MT, USA; ^2^Faculty of Medicine, Department of Basic Neurosciences, University of GenevaGeneva, Switzerland

**Keywords:** electrophysiology, microelectrode, non-human primate, methods, spatiotemporal activity, large-scale recordings

## Abstract

Cognitive processes play out on massive brain-wide networks, which produce widely distributed patterns of activity. Capturing these activity patterns requires tools that are able to simultaneously measure activity from many distributed sites with high spatiotemporal resolution. Unfortunately, current techniques with adequate coverage do not provide the requisite spatiotemporal resolution. Large-scale microelectrode recording devices, with dozens to hundreds of microelectrodes capable of simultaneously recording from nearly as many cortical and subcortical areas, provide a potential way to minimize these tradeoffs. However, placing hundreds of microelectrodes into a behaving animal is a highly risky and technically challenging endeavor that has only been pursued by a few groups. Recording activity from multiple electrodes simultaneously also introduces several statistical and conceptual dilemmas, such as the multiple comparisons problem and the uncontrolled stimulus response problem. In this perspective article, we discuss some of the techniques that we, and others, have developed for collecting and analyzing large-scale data sets, and address the future of this emerging field.

## Introduction

Decades of microelectrode recordings have laid the foundation for our current understanding of cognitive processes at the single neuron level, while functional imaging and other non-invasive techniques have demonstrated that cognitive processes engage widespread cortical and subcortical areas (Fuster and Alexander, 1971; Andersen et al., 1985; Moran and Desimone, 1985; Funahashi et al., 1989; Chelazzi et al., 1993; Desimone and Duncan, 1995; Courtney et al., 1997; Munk et al., 2002; Constantinidis and Procyk, 2004; Postle, 2006; Harrison and Tong, 2009; Roux and Uhlhaas, 2014). These studies, along with much anatomical and theoretical work, have lead to a common agreement that cognitive processes must involve the dynamic, self-organized coordination of multiple cortical and subcortical areas (Mesulam, 1990; Gray, 1994; Bressler, 1995; Friston, 1997; Bressler and Kelso, 2001; Varela et al., 2001; Fries, 2005; Buzsáki, 2010; Chialvo, 2010; Siegel et al., 2012; Singer, 2013; Tognoli and Kelso, 2014). Understanding how populations of neurons distributed across the brain coordinate their activity in relation to behavior has become a central question in cognitive and systems neuroscience. As the title of this research topic suggests, studying the functional connectivity patterns that arise during cognitive processes may be the key to answering this question.

In order to properly study large-scale brain dynamics, recording technologies with the requisite coverage, and spatiotemporal resolution must be developed. Current techniques make tradeoffs between coverage, spatial resolution, and temporal resolution. For instance, functional MRI (fMRI) indirectly measures neural activity on the time scale of seconds, but can sample signals from small contiguous volumes spanning the entire brain. Standard single microelectrode recordings on the other hand, provide unparalleled spatiotemporal resolution, but poor coverage. The signals recorded with other technologies, such as Electroencephalography (EEG), Magnetoencephalography (MEG) and Electrocorticography (ECoG), also vary substantially in their coverage and spatial resolution, with clear limitations in one or more variables. These severe methodological constraints have prompted several groups, including our own, to develop large-scale microelectrode recording devices, which have high coverage and both high spatial and high temporal resolution. What follows, is a discussion of the state of the art in large-scale microelectrode recordings in the non-human primate.

## Large-Scale Recording Systems in the Non-Human Primate

Recording technologies for primates and other mammals allow for recording from entire networks, localized circuits, or a combination of the two (Maynard et al., 1997; Buzsáki, 2004; Kipke et al., 2008; Chang, 2015; Lewis et al., 2015). We restrict our discussion to techniques that use microelectrodes or microwires to chronically or semi-chronically record local field potentials (LFP) and/or unit activity from multiple distributed sites simultaneously. Unfortunately, many interesting studies and methods are left out solely for the purpose of brevity or because they are outside our narrow focus (for example, see Bosman et al., 2012; Yanagawa et al., 2013; Mendoza-Halliday et al., 2014; Bastos et al., 2015; Siegel et al., 2015).

Early examples of large-scale recordings in the non-human primate include the work from Bressler et al. (1993), Nicolelis et al. (1998, 2003) and Hoffman and McNaughton (2002). Using chronically implanted surface-to-depth bipolar electrodes, Bressler et al. (1993) recorded transcortical LFP from up to 15 broadly distributed sites in macaque monkeys performing a visual pattern discrimination task. Electrodes were implanted in frontal, motor, somatosensory, superior and inferior temporal, parietal, pre-striate and striate areas (Bressler et al., 1993). This study revealed broadband coherence between distinct combinations of widely separated sites that occurs in a task-dependent manner. Also using chronic recording methods, Nicolelis et al. (1998) implanted three microwire arrays in three different cortical areas (area 3b, area S2, and area 2) in owl monkeys to identify the tactile information contained in neural populations. Each array, arranged in a 2 by 8 matrix, sampled an area of 2 mm^2^ using 16 stainless steel, teflon-coated wires. This was later scaled up to over 700 microwires in 10 cortical locations over both hemispheres using stacks of microwire arrays directly attached to connectors (Nicolelis et al., 2003). Animals in this study were implanted for up to 18 months, with early recording sessions boasting up to 247 single neurons.

While these landmark studies made important scientific and technical advances, the recording techniques still had several drawbacks. An ideal large-scale recording device would have independently moveable electrodes and the ability to record from both cortical and sub-cortical areas. Hoffman and McNaughton (2002) overcame the first limitation by chronically implanting arrays with 144 independently moveable electrodes in four different cortical regions (dorsal prefrontal cortex, somatosensory cortex, posterior parietal cortex, and motor cortex) in a macaque monkey performing a sequential reaching task. Electrodes were densely spaced at 650 micron intervals in a 12 by 12 matrix. Using these high-density distributed recordings they provided evidence for coordinated reactivation occurring within and between all of the recorded regions except for dorsal prefrontal cortex. Similarly, Schwarz et al. (2014) modified their earlier design (Nicolelis et al., 2003) to allow for their microwire arrays to be repositioned, along with increasing the channel count and incorporating a telemetry system.

Sub-cortical recordings are also crucial for an accurate understanding of large-scale brain dynamics. Even though the previously mentioned techniques have made major advances in one way or another, they all lacked the ability to make sub-cortical recordings. In part, this is probably because that was not the objective of the experiments. Recognizing the importance of sub-cortical recordings, Feingold et al. (2012) developed a system capable of both cortical and sub-cortical recordings using independently moveable microelectrodes. Using this device, they were able to make simultaneous recordings from 14 different brain regions using up to 127 microelectrodes. This study provides an excellent example of a system with immense potential for studying large-scale brain dynamics.

**Figure 1:**
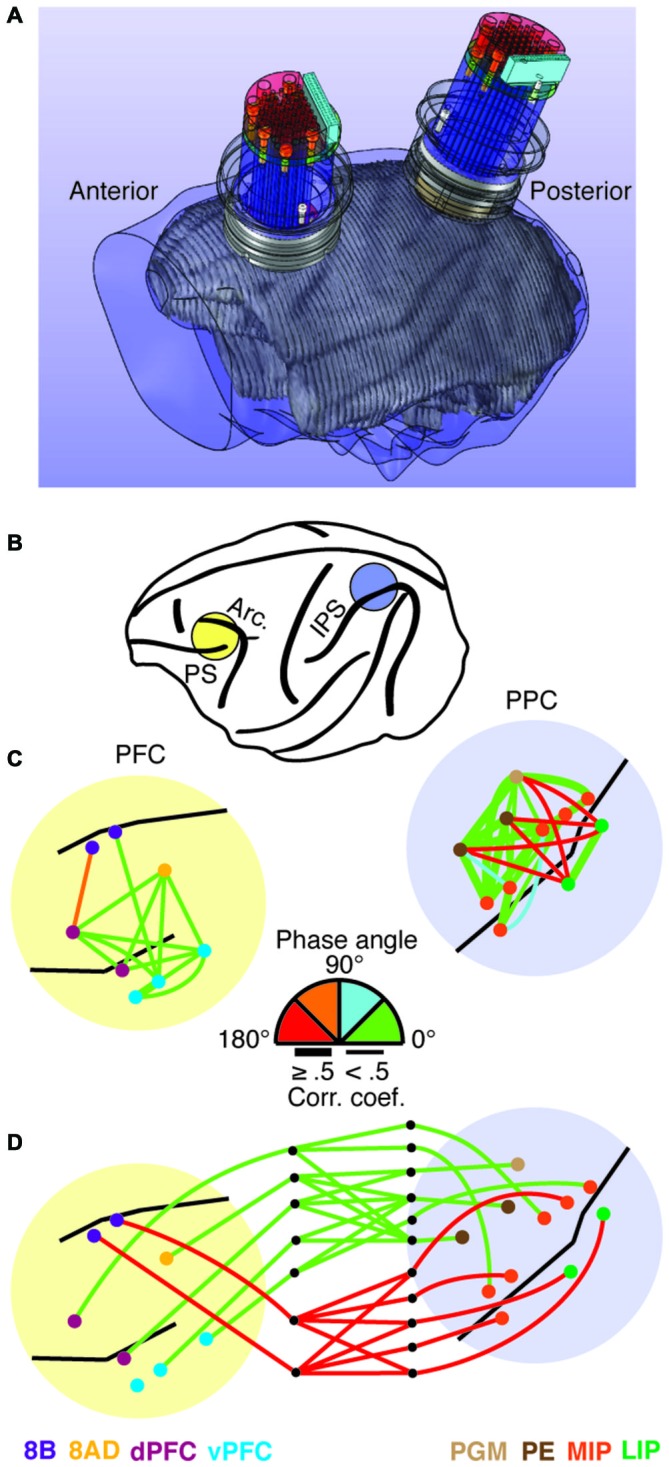
**Illustration of the hardware for simultaneous frontoparietal recordings and an example of the relative phase relationships in the frontoparietal network. (A)** MRI based skull and brain model incorporating 3D design drawings of two 32 channel semi-chronic microdrives and recording chambers. **(B)** Illustration of the recording chamber locations. **(C,D)** Magnitude and phase angle within and between prefrontal and posterior parietal cortex during the delay period of a visual working memory task. The magnitude of correlation is indicated by line thickness, and the relative phase angle (absolute value) is indicated by the line color. The relative phase angles were calculated using cross-correlation on bandpassed signals (8–25 Hz). **(C)** Intra-prefrontal and intra-parietal relative phase angles. Note the clear anti-phase relationship between signals on opposite sides of the intraparietal sulcus. **(D)** Inter-areal relative phase angles. In this example, a clear anti-phase relationship exists between prefrontal area 8B and the parietal areas it is significantly correlated with (see Dotson et al., 2014 for more details).

Over the past decade, we have been developing large-scale recording devices as well. Our objective has been to develop a technique capable of both cortical and sub-cortical exploration, along with the ability to move electrodes independently and scale the devices as needed. Devices developed in the lab include semi-chronic microdrives with 32 or 60 independently moveable microelectrodes capable of simultaneously recording the activity from multiple cortical areas (Gray and Goodell, 2011), to devices with over 250 microelectrodes capable of recording from an entire cerebral hemisphere. The 32-channel microdrive was developed for targeting several nearby cortical areas at a variety of depths. Electrodes are arranged in a 6 by 6 grid (one of the rows is only partially filled) and horizontally spaced at 1.5 mm. Each electrode is independently moveable, with up to 16 mm of travel (for more details, see Gray and Goodell, 2011; Markowitz et al., 2011; Salazar et al., 2012). Figure 1A shows design drawings of two of these devices integrated into an MRI reconstruction of a macaque skull and brain. The recording devices are semi-chronically implanted, meaning they may be removed at the end of a study or replaced with new ones. We used these devices to simultaneously record from a total of 12 different cortical areas in prefrontal and posterior parietal cortex in an animal performing a visual working memory task (Figure 1B; Salazar et al., 2012; Dotson et al., 2014). Using this recording technique, we were able to identify a bimodal patterning of the relative phase relationships among cortical signals (LFPs 8–25 Hz) within the frontoparietal network (Dotson et al., 2014). Figures 1C,D shows an example of the magnitude and relative phase relationships within and between prefrontal and posterior parietal signals during visual working memory from a single recording session (for more details, see Dotson et al., 2014). The relative phase angle was estimated using cross-correlation analysis on pairs of signals bandpass filtered at 8–25 Hz.

**Figure 2:**
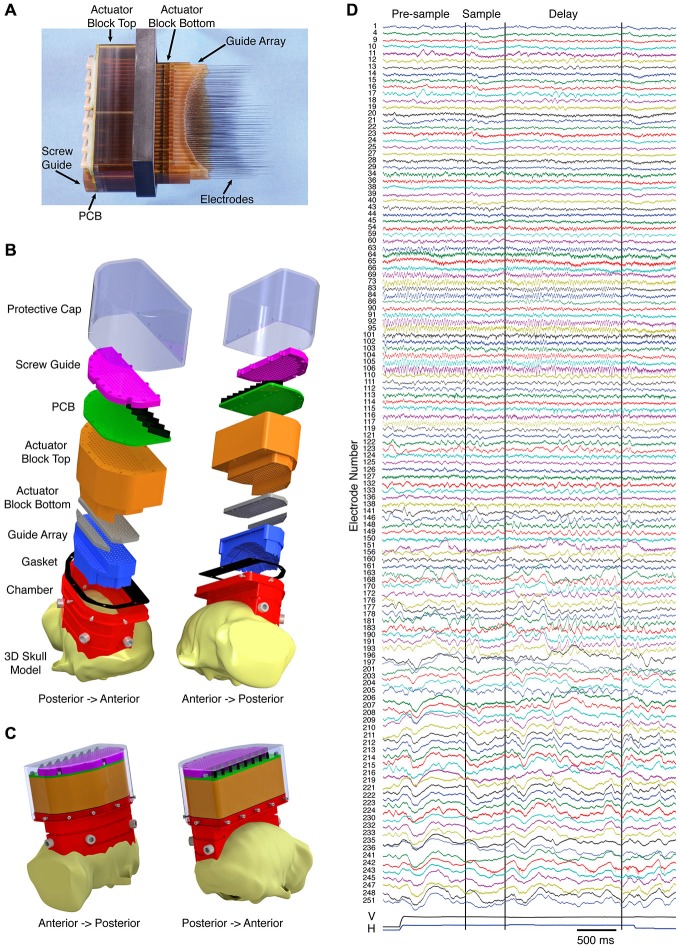
**Hemisphere-wide recording technique. (A)** Semi-chronic 252-channel microdrive system with electrodes advanced. Prior to implantation, the electrodes are fully retracted and the bottom surface of the device is made impermeable. **(B)** Exploded views of the design drawing for the microdrive system (the actuator mechanism is excluded). **(C)** Design drawings of the system as it would appear when implanted on the animal. **(D)** Example of the broadband signals (local field potentials (LFP) and single/multi-unit activity) simultaneously recorded from 127 electrodes on a single trial of a visual working memory task. From top to bottom the signals go from anterior to posterior, respectively. The vertical (V) and horizontal (H) eye positions are included at the bottom of the plot. The first two vertical black lines indicate the onset and offset of the sample. The third vertical black line indicates the onset of the match.

While the use of multiple 32-channel microdrives aided us in making parallel recordings in the frontoparietal network, our ultimate objective has been to simultaneously record from the entire working memory network, including the dorsal and ventral visual streams, the prefrontal cortex, and subcortical areas, such as the mediodorsal nucleus of the thalamus. Using two macaque monkeys trained to perform a visually guided delayed match to sample task, we implanted recording devices with over 250 independently moveable microelectrodes spanning an entire hemisphere. Figure 2A shows a side view of the fully loaded device (second generation, 252 electrodes), with electrodes protruding. The electrodes are spaced at 2.4 mm intervals and have a travel of 40 mm on the most lateral three columns and 30 mm on the remaining columns. Prior to implantation, the electrodes are retracted, and the whole device is hermetically sealed using silicone grease followed by a silicone sealant. The implant is done in three steps over a period of several months. First, a chamber is mounted on the skull using cranial screws and bone cement. Obtaining a tight fit between the chamber and the skull is accomplished by customizing the chamber to the animal’s skull using a pre-operative MRI, and by applying bone cement to the interface between the chamber and the skull. Next, a craniotomy is made within the confines of the chamber, and finally the sealed 252 channel microdrive is placed into the chamber. Importantly, the recording device is designed to conform to the surface of the dura, essentially replacing the bone, which assures very little movement of the brain. Figure 2B shows exploded design drawings of the microdrive without actuator components (note: craniotomy is not shown). Figure 2C shows the device, as it would appear fully assembled and implanted. Using these devices, we have recorded high quality broadband signals (LFP and single/multi-unit activity) from months to nearly a year, with the ability to target dozens of cortical and subcortical areas simultaneously. Figure 2D shows an example of the broadband data recorded on a single trial from 127 electrodes. From top to bottom the signals go from anterior to posterior, respectively, and span prefrontal cortex to primary visual cortex. Note the differences in spectral composition and task dependence.

## Caveats with Large-Scale Recordings

There are many hurdles to overcome when running large-scale microelectrode experiments in non-human primates, on top of the already difficult task of training, implanting, and recording from an alert animal. For instance, the data sets are several orders of magnitude larger than ones collected using single electrodes and subsequently require the use of large servers. Computational demands also increase by many orders of magnitude, especially when performing pairwise or multivariate measures. This means more resources, monetary and human, are necessary to complete these projects. Researchers must also become savvy programmers in order to utilize high performance computers and to write computationally efficient programs. Here, we mention a few of the issues that arise during the analysis phase. Specifically, the multiple comparisons problem and what may be referred to as the uncontrolled stimulus response problem.

Analyzing data from large-scale recordings often requires performing multiple parallel statistical tests, leading to the multiple comparisons problem (Shaffer, 1995; Benjamini, 2010). Generally speaking, this issue arises because the probability of making a Type 1 error (false positive) goes up as the number of tests goes up. While the concept is relatively easy to grasp, many interesting debates have arisen around it. For instance, where does one draw the border for a family of tests (Rothman, 1990; Perneger, 1998)? Perneger (1998) takes this issue to an extreme in a discussion of the Bonferroni correction by asking if corrections should be made for all the tests a researcher performs in their career, or if all the tests in a particular journal should be corrected. Misinterpretation, or exclusion, of findings due to high Type 2 error rates (false negative) are also a concern, leading some researchers to advocate against correction procedures altogether (Rothman, 1990). With that said, a commonly accepted technique that balances both type 1 errors (false positive) and type 2 errors (false negative) is to control the false discovery rate (FDR). FDR procedures, like the Benjamini and Hochberg (1995) procedure, ensure that only a certain percentage of Type 1 errors occur (e.g., 5%) and rely on the assumption that a small proportion of Type 1 errors will not alter the conclusions of the study. In fMRI research, FDR procedures, initially introduced by Genovese et al. (2002), have been widely used. Indeed, we can learn a lot from fMRI researchers, who often make 1,000 s of parallel tests, and have been hashing these issues out for many years (Logan and Rowe, 2004; Bennett et al., 2009; Chumbley and Friston, 2009; Nichols, 2012). EEG and MEG researchers have also developed techniques to deal with the multiple comparisons problem, including nonparametric and cluster-based nonparametric tests (Maris and Oostenveld, 2007). Large-scale microelectrode recordings will inevitably be another hot bed for the multiple comparisons problem. Subsequently, it will be important that researchers take the time to understand how results should be interpreted based on different correction procedures, and to avoid focusing too closely on the arbitrary designation of “significance”. Tukey (1991) puts this wisely: “We must pay some chance of error to extract knowledge—or belief—from data. A crucial task is to expend this chance wisely, and to see that how it was spent, as well as how much was spent, is clearly recognizable”. It should also be stressed that since scientific progress relies on large bodies of work there is an inherent control for the multiple comparisons problem: reproducibility (Tukey, 1991; Perneger, 1998).

Methodological issues in study design and interpretation of results also arise when moving from the analysis of single neurons to analyses that bridge mesoscopic and macroscopic levels. Typical microelectrode experiments involve placing a single electrode in a predefined area of the brain, identifying a neuron with desirable response properties, and then tailoring the experimental paradigm for that specific neuron. This provides the ideal setup for understanding the response properties of single neurons in single areas to highly controlled changes in stimulus and task properties, and it has provided us with the foundation of cognitive and systems neuroscience. However, gaining a better understanding of cognitive processes will rely on both an understanding of the response properties of single neurons, and by determining how many widely distributed cortical and subcortical areas cooperate in a dynamic circuit. The brain is after all a complex system, and should be studied as such (Chialvo, 2010; Gazzaniga, 2010). Unfortunately, when the functions of large-scale circuits are of interest, it becomes impractical to search for recording sites in many locations with overlapping response properties (Herrington and Assad, 2010). Moreover, tailoring experiments to only one or two recording sites out of 10’s to 100’s of recordings sites would also be impractical. How then should simultaneous recordings from two or more areas be conducted? The approach that we (Salazar et al., 2012; Dotson et al., 2014) and other researchers (Buschman and Miller, 2007; Pesaran et al., 2008; Siegel et al., 2015) have taken is to determine the response properties of neurons *post hoc*, rather than selecting neurons with specific response properties. However, these differences in study design can place researchers in a position where their results and interpretations are not able to entirely overlap with previous work in the field. Indeed, significant debate has arisen along these lines (Miller and Buschman, 2007; Schall et al., 2007). From this, it may be clearly seen that in order to record the distributed patterns of activity involved in perception, action, and everything in between, we must adjust our analysis of single neuron response properties. This is especially true if we hope to understand behavior in naturalistic or real world settings. We foresee that as more data is collected, new avenues of investigation that do not rely as closely on single neuron properties will open up, but until then, understanding the tradeoffs and how this affects study design and interpretation of results will be crucial to the success of these types of studies.

## The Future of Large-Scale Recordings in the Non-Human Primate

Large-scale microelectrode recordings in the non-human primate are beginning to be paired with common manipulation techniques, such as electrical stimulation and pharmacological injections (Feingold et al., 2012). Future work could include injecting agonists or antagonists for neurotransmitters like dopamine and acetylcholine, which have been shown to play a central role in attentional processes in prefrontal and visual cortex, respectively (Herrero et al., 2008; Noudoost and Moore, 2011a,b). By combining pharmacological injections with simultaneous recordings from the injection sites and other cortical and subcortical areas distributed across the brain, the contribution of these neurotransmitters to cognitive processes could be further understood. Similarly, large-scale microelectrode recording devices could incorporate emerging technologies, such as optogenetic tools developed specifically for non-human primates (Han et al., 2009; Diester et al., 2011; Han, 2012; Gerits and Vanduffel, 2013; Dai et al., 2014). Combining optogenetics with large-scale microelectrode recordings will enable researchers to selectively manipulate and essentially dissect any circuit of interest (Gerits et al., 2012). Chemogenetic tools, such as designer receptors exclusively activated by designer drugs (DREADDs; Sternson and Roth, 2014; Vardy et al., 2015), which are in the early stages of development for non-human primates, provide another promising tool to use in conjunction with large-scale recordings.

Finally, the future of large-scale microelectrode recordings will also rely on the utilization of both functional connectivity measures (this research topic, see Bastos et al., 2015) and machine learning tools (for a recent review, see Jordan and Mitchell, 2015). Machine learning tools, like multi-voxel/variate pattern analysis (MVPA), have already converted much of the functional imaging community from focusing on functional localization to focusing on patterns of activity (Harrison and Tong, 2009; Pereira et al., 2009; Poldrack, 2012; Rissman and Wagner, 2012). The multi-variate data produced by large-scale microelectrode recordings combined with machine learning tools will help electrophysiologists make the same transition. Ultimately, better understanding of the large-scale activity patterns underlying behavior will be achieved by combining several of these emerging technologies and analytical techniques with large-scale microelectrode recordings.

## Author Contributions

The ideas and content within this article were put together through a collaborative effort by all authors (NMD, BG, RFS, SJH and CMG).

## Funding

This work was supported by National Institute of Mental Health Grant MH081162 (CMG), National Institute of Neurological Disorders and Stroke Grant NS059312 (CMG), a McKnight Memory and Cognitive Disorders award (CMG), the Swiss National Science Foundation (RFS), and the Kopriva Foundation (NMD).

## Conflict of Interest Statement

The authors declare that the research was conducted in the absence of any commercial or financial relationships that could be construed as a potential conflict of interest.
